# Milk progesterone enzyme-linked immunosorbent assay as a tool to investigate ovarian cyclicity of water buffaloes in relation to body condition score and milk production

**DOI:** 10.1186/1751-0147-54-30

**Published:** 2012-05-03

**Authors:** Turgish A Banu, Mohammed Shamsuddin, Jayonta Bhattacharjee, Mohammad F Islam, Saiful I Khan, Jalal U Ahmed

**Affiliations:** 1 Department of Surgery and Obstetrics, Faculty of Veterinary Science, Bangladesh Agricultural University, Mymensingh, 2202, Bangladesh

**Keywords:** Milk, Progesterone, ELISA, Cyclicity, Body condition score, Buffalo

## Abstract

**Background:**

Application of assisted reproductive technologies in buffaloes is limited to some extent by farmers’ inability to detect oestrus because of its poor expression. The present study aimed at investigating reliability of a milk progesterone enzyme-linked immunosorbent assay (ELISA) to assess the ovarian cyclicity during post partum, oestrus and post-breeding periods in water buffaloes.

**Methods:**

Progesterone concentrations were measured by an ELISA in milk of 23 postpartum buffaloes in relation to oestrus, pregnancy, body condition score (BCS) and milk production. Two milk samples were taken at 10 days intervals, every month starting from day 30 and continued to day 150 post partum. BCS and milk production were recorded during sample collection. Milk samples from bred buffaloes were collected at Day 0 (day of breeding), Days 10–12 and Days 22–24. Defatted milk was preserved at −80°C until analysis. Pregnancy was confirmed by palpation per rectum on Days 70–90.

**Results:**

Seventeen buffaloes had 47 ovulatory cycles, one to four in each, 13 were detected in oestrus once (28 % oestrus detection rate). Progesterone concentration ≥1 ng/ml in one of the two 10-day-interval milk samples reflected ovulation and corpus luteum formation. The intervals between calving to first luteal activity and to first detected oestrus varied from 41 to 123 days (n = 17) and 83 to 135 (n = 13) days, respectively. Eight buffaloes were bred in the course of the study and seven were found pregnant. These buffaloes had a progesterone profile of low (<1 ng/ml), high (≥ 1 ng/ml) and high (≥ 1 ng/ml) on Day 0, Days 10–12 and Days 22–24, respectively. Buffaloes cycling later in the postpartum period had fewer missed oestruses (*P* < 0.05). Buffaloes with a superior BCS had a shorter calving to oestrus interval and produced more milk (*P* < 0.05).

**Conclusions:**

Milk progesterone ELISA is a reliable tool for monitoring ovarian cyclicity and good BCS may be an indicator of resuming cyclicity in water buffalo.

## Background

Reproductive management of buffaloes is a major concern because of difficulties in oestrus detection and is associated with significant economic losses. Shah *et al.*[1] reported a 6-73 % incidence of silent oestrus or suboestrus in buffaloes. This warrants development and application of technologies to identify silent oestrus in buffaloes. It is also important for the farmers to understand the importance of body condition scoring (BCS) and its association with ovarian cyclicity and to ask for veterinary services when buffaloes do not exhibit behavioural oestrus.

Milk progesterone concentration is a good predictor to determine the functional status of the corpus luteum and ovarian activity in domesticated ruminants [2]. Presicce *et al.*[3] used plasma progesterone concentration measurement along with ultrasonography to monitor ovarian follicular dynamics in Mediterranean Italian buffaloes and in a recent study using plasma progesterone concentration measurement along with rectal palpation, 50 % of buffaloes suspected of having anoestrus were actually in suboestrus [4]. Milk is preferred over plasma for progesterone concentration determination under field conditions, because milk is easy to collect and ultimately ensure farmers compliance. Many of the earlier studies measured milk progesterone by radioimmunoassay or enzyme immunoassay. But use of a milk progesterone enzyme-linked immunosorbent assay (ELISA) with an appropriate sampling programme and its application in the field to monitor ovarian cyclical status in buffaloes remains to be elucidated. Monitoring milk progesterone concentration gives practical information to improve reproduction and to detect reproductive disorders associated with ovarian dysfunction [5,6]. Rectal palpation can be used to detect some ovarian disorders in buffaloes but the findings not always correlate with ovarian activity [7]; findings that could be due to corpora lutea being embedded in the ovarian stroma particularly during the early post partum period in buffalo cows [8]. Ultrasonography could be an option to solve this issue but its use in smallholder farms is limited by the cost. Therefore milk progesterone ELISA with appropriate sampling remains as the most applicable tools of assessing ovarian cyclical status in buffaloes under field conditions.

BCS is a useful indicator of nutritional status and good BCS reflecting positive energy balance leads to earlier resumption of postpartum ovarian cyclicity in cows [9,10]. Therefore it is important to investigate the relationship of BCS with the resumption of ovarian cyclicity and milk production in dairy buffaloes in Bangladesh. This will help educating farmers on predicting the ovarian cyclicity and planning breeding of post partum buffaloes.

The present study aimed at investigating the ovarian cyclicity using milk progesterone ELISA in water buffaloes during the postpartum, oestrus and post mating periods and to relate progesterone levels with BCS, milk production and pregnancy diagnosis by palpation per rectum.

## Methods

Twenty three buffaloes from 23 farms located in six villages of the Mymensingh District, Bangladesh were selected. The study was conducted during the period from June 2006 to March 2007. Each family owned 1 or 2 buffalo cows of the indigenous river type. The age ranged from 4 to 15 years and parity ranged between 0 and 7. The buffaloes were fed on rice straw, cut-and-carry grasses and 0.5 to 1.5 kg milling by-product as concentrate (rice polish and/or mustered oil cake) with limited grazing on roadside and community land. Buffaloes were selected on the basis of similar feeding and management pattern. The animals were monitored regularly to ensure that they were not stressed thus limiting variation of circulating hormone levels [11].

### Body condition scoring

Buffaloes were registered two months before parturition and age, breed, parity, and feeding management information was recorded. Body condition of the buffaloes was scored by using a 1–5 scale (1 = thin and 5 = obese) [12,13]. Photographs were taken from lateral and rear views of each buffalo to retrospectively determine the BCS. A team of four members evaluated the photographs and scored BCS. BCSs were scored at eight months of gestation, at calving and at the time of every milk sample collection.

### Milk sample collection and preservation

Milk samples were collected directly from the teats of individual buffaloes at the middle of morning milking into a screw-capped plastic tube containing 2-bromo-2-nitro-1, 3-propanediol (Bronopol 8 mg tablet/ 40 ml of milk, D&F Control Systems Inc. Dublin, CA, USA). Milk sampling was started 30 days post partum and two samples at 10 days interval were collected per month up to 150 days post partum if oestrus was not detected. Farmers were asked, if they saw any signs of oestrus *viz*. restlessness, bellowing, vulval swelling, mucous discharge, frequent voiding of small quantities of urine. At every farm visit for milk sampling, the milk production and signs of previous oestrus, i.e. dry or fresh vaginal discharge adhered to the perineum or tail, were recorded. Once detected in heat, the animals were bred by natural mating. Thereafter three additional milk samples were collected. The first one was on Day 0 (day of breeding) while the second and third samples were collected on Days 10–12 and Days 22–24, respectively. Whole milk samples were stored at 4°C for up to two days. The samples were defatted by centrifugation at 1500 g for 30 min at 4°C, the fat-free milk portions were collected and preserved at −80°C until analysed by ELISA.

### Preparation of progesterone standards for ELISA

Two-litre fresh milk from a buffalo cow in oestrus was collected and defatted. Activated charcoal (Sigma Aldrich, St. Louis, MO, USA) was added (0.25 % w/v) and the milk was stirred one hour at room temperature (around 22°C) and then preserved overnight at 4°C. The charcoal-added milk was centrifuged for 15 min at 1500 g at 4°C, the supernatant was collected and the residual charcoal was filtered by filter paper. Repeated centrifugation and filtration were performed to remove charcoal completely. Then Bronopol was added as a preservative (8 mg/40 ml of milk). Standard solutions of defatted milk containing 100, 10, 5, 2.5, 1.25, 0.63, 0.31 and 0.16 ng/ml progesterone were prepared by using commercially available progesterone (Calbiochem, Biosciences Inc. CA, USA). This procedure was done immediately before adding samples and standards in the microtitre plate.

### Milk progesterone ELISA

Milk progesterone concentration was determined through a competitive ELISA following the protocol proposed by Rasmussen *et al.*[14] with minor modifications. Briefly, a 96 well plate (Nunc Immuno plate, Roskilde, Denmark) was coated and incubated overnight at 4°C with 100 μl of a 2 μl/ml secondary antibody (Goat anti-mouse antibody, Calbiochem) in coating buffer (0.05 M carbonate-bicarbonate buffer, pH 9.6). Then the plate was washed four times with wash buffer (0.04 M 3-[N-Morpholino] propanesulfonic acid with 0.05 % Tween20; pH 7.2). One hundred μl of anti-progesterone monoclonal antibody solution (1:40,000 dilution; IAEA, Vienna, Austria) in assay buffer (0.04 M 3-[N-Morpholino] propanesulfonic acid, 0.12 M sodium chloride, 0.01 M EDTA, 0.05 % Tween20, 0.005 % chlorohexidinedigluconate digluconate, and 0.1 % gelatin; pH 7.4) was added to each well of the plate except nonspecific binding wells and incubated with shaking for 1.5 h at room temperature. Thereafter the plate was washed four times with wash buffer, and 100 μl of standards, quality control samples (5 ng progesterone/ml of skimmed and charcoal stripped milk) and milk samples to be tested were added to the plate and incubated with shaking for 1.5 h at room temperature. Fifty μl of horseradish peroxidase (HRP) conjugate (1:2000 in assay buffer; Progesterone-3-HRP produced from progesterone-3-CMO, Steraloids Inc., Newport, RI, USA) was added. The plate was shaken again for 1.5 h and washed with wash buffer four times. Freshly prepared 125 μl of 3, 3', 5, 5'-tetramethylbenzidine substrate was added and shaken for 15 min at 37°C in dark to quantify HRP activity. The reaction was stopped by adding 50 μl stop solution (0.5 M Sulphuric acid) and read by an automatic microplate reader (Microplate spectophotometer, Molecular Device, Sunnyvale, CA, USA) at a predefined setting (primary wave length 450 nm, reference wavelength 600 nm, end point). The optical density (OD) values were analyzed by a pre-customized MS Excel work sheet 2002 to determine the progesterone concentration [15]. The intra-assay coefficient of variances ranged from 2.2 % to 9.5 % (4 wells) and interassay coefficient of variance was 9.7 % (5 assays).

### Pregnancy diagnosis

The genital organs of buffaloes were examined per rectum for pregnancy diagnosis between Day 70 and Day 90 after natural mating.

### Statistical analysis

The data on milk progesterone concentration, milk production and BCS against months and days of sampling were presented graphically by using Microsoft Excel. Graphs were prepared with data on milk progesterone concentration, milk production and BCS against months and days of sampling. A progesterone concentration of ≥1.0 ng/ml of skim milk was referred to as a progesterone rise, due to a functional corpus luteum [9,16-19]. When a progesterone rise above 1 ng/ml was detected in one or both samples collected at 10 days interval, it was considered that the buffalo had ovulated unnoticed by the farmer (missed oestrus). A progesterone concentration of less than 1.0 ng/ml was considered as a low level, indicating absence of a functional corpus luteum [20]. Oestrus detection rate (EDR) was calculated by the following equation [21]:

(1) $$\text{EDR}=\frac{\text{Number of detected oestruses}}{\text{Number of detected oestruses}+\text{number of missed oestruses}}\times 100$$

Intervals between calving and first detected oestrus, calving and first detected progesterone peak (an indicator of earlier ovulation) and calving and conception were calculated. Percentages of buffaloes not detected in oestrus and non-cyclic buffaloes during the study period were calculated. Regression analysis was performed to determine the relationship of BCS at calving and oestrus with the calving to first oestrus interval and with milk production [22].

## Results

**Figure 1:**
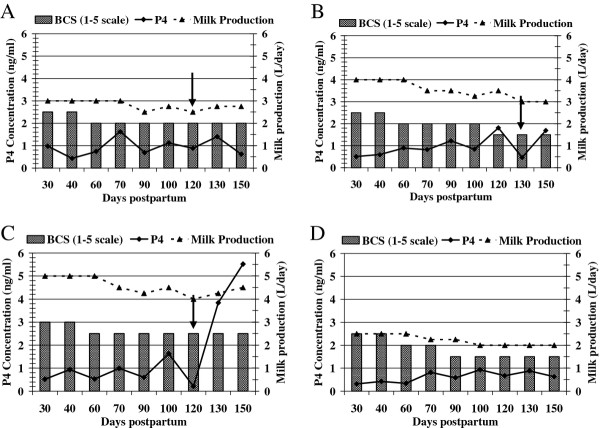
Body condition score, progesterone concentrations in milk and milk production of buffalo cows showing luteal activity at different times in postpartum period.

**Figure 2:**
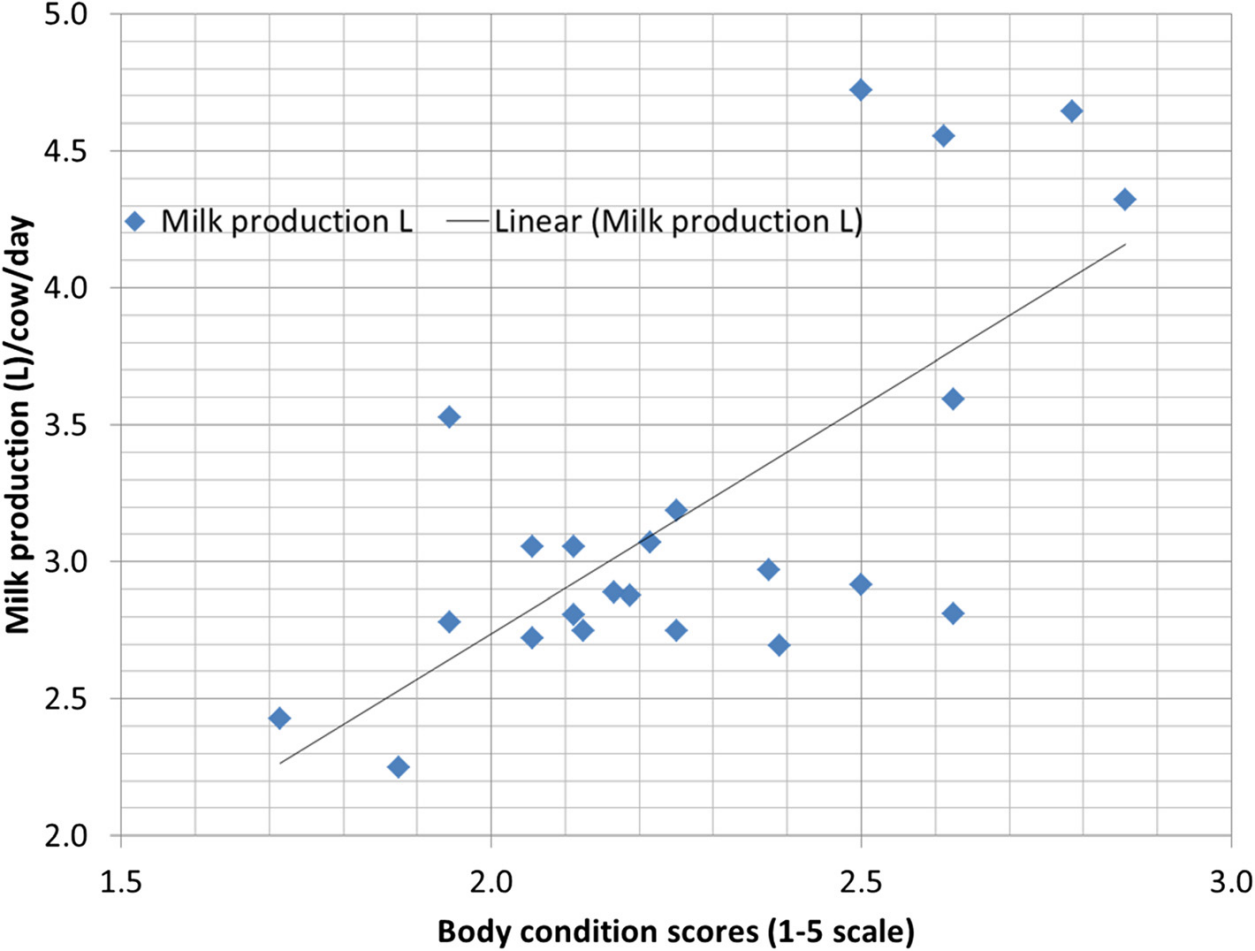
Relationship between milk production (L) and body condition score (1–5 scale) of buffalo cows on the day of farm visit twice a month at 10 days interval, starting from Day 30 through Day 150 or to the detection of first oestrus postpartum.

BCS of buffaloes studied ranged from 1.5 to 3.5 at the time of oestrus detection. Figures 1A-C show data on BCS, milk progesterone concentrations and milk production of three buffalo cows being representative for cows showing luteal activity at 30–70, 71–90 and 91–130 days post partum, respectively. Figure 1D shows data of a buffalo typical for cows that remained acyclic through 150 days post partum. All buffaloes showed a trend of lower BCS with progression of post partum period. Most of the buffaloes gradually reduced milk yield with loss of BCS. A significant (*P* < 0.001) linear relationship existed between the post partum BCS and milk yield (Figure 2) with increased milk production being associated with higher BCS.

In cyclic buffaloes, the intervals between calving to first luteal activity and first detected oestrus were 41 to 123 (n = 17) and 83 to 135 (n = 13) days, respectively.

Assuming that each progesterone rise above 1 ng/ml in milk followed an ovulation and a normal cycle, 47 cycles occurred in 17 buffaloes. Oestrus was detected visually in 13 cows while 6 buffaloes remained acyclic through 150 days postpartum. This leads to a 28 % oestrus detection rate. Farmers missed in average 1.8 oestruses per buffalo. The average number of missed oestrus in Day 30–70 post partum buffaloes (2.55) was significantly (*P* < 0.05) higher than those of Day 71–90 (1.33) and Day 91–130 (0.65) postpartum buffaloes. When farmers missed more oestrous cycles, the length of post partum period (calving to the first detected oestrus) increased (Table 1).

**Table 1:** Missed oestrous cycles and calving to first oestrus intervals in post partum buffaloes

**Number of missed oestrous cycles**	Number of buffaloes*	**Average calving to first detected oestrus interval (days) (Mean ± SEM )**
0	3	96 ± 8
1	4	99 ± 8
2	6	121 ± 5
3	2	Not detected in oestrus
4	2	Not detected in oestrus

* 17 buffaloes had a total of 47 ovulatory cycles.

**Figure 3:**
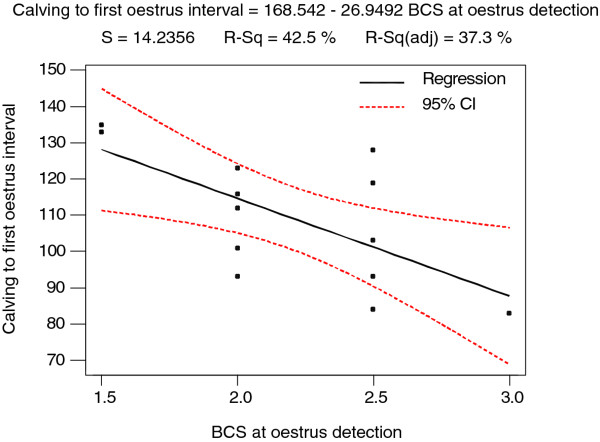
Relationship between body condition score at oestrus detection and calving to first oestrus interval in buffalo cows.

Buffaloes with higher BCS at the time of oestrus detection had shorter interval between calving and first detected oestrus than those with lower BCS (Figure 3; *P* < 0.05). However, the BCS scored during the gestation period and at calving did not show any significant effect on the intervals between calving to first rise of milk progesterone and between calving and first detected oestrus.

Only 8 buffaloes were bred during the study period. Seven buffaloes had a progesterone profile being low (<1 ng/ml), high (≥1 ng/ml) and high (≥1 ng/ml) on Day 0, Days 10–12 and Days 22–24, respectively. These buffaloes were found pregnant on rectal palpation of the genital tract between Day 70 and Day 90. The buffalo with progesterone profiles being low, high and low on Day 0, Days 10–12 and Days 22-24, respectively, returned to oestrus. In pregnant buffaloes, the milk progesterone concentrations (mean ± SEM ) at Days 22–24 (4.8 ± 0.4 ng/ml) were significantly (*P* < 0.001) higher than that at Days 10-12 ( 2.0 ± 0.4 ng/ml).

## Discussion

Our protocol and sampling regime for milk ELISA detected a large proportion (74 %) of buffaloes that had resumed their ovarian cyclicity by Day 150 post partum but the farmers’ oestrus detection rate was low under the assumption that the observed progesterone rise in milk followed an ovulation and a normal cycle. In accordance with these findings, Barkawi *et al.*[23] showed that approximately 90 % of the buffaloes resumed their ovulatory cycle within 60 days post partum. The reasons for missed oestrus could be due to silent ovulation or anovulatory luteinization of follicles during the early postpartum period [24-26]. However, differences in production system and breeds of animals might have resulted in the variation in post partum ovarian activity among the published studies. Usmani *et al.*[27] found that 86 % of Nili-Ravi buffaloes showed at least one short luteal phase 8 to 13 days before the first oestrus. Accordingly, data of the present study indicate a higher number of missed oestrus in buffaloes that resumed luteal activity between Day 30 and Day 70 than those resuming luteal activity between Day 71 and Day 130. It remains to investigate to which extent the first post partum ovulation is associated with oestrus signs in some buffaloes. Extension programmes on educating farmers on oestrus behaviour and heat detection based on secondary signs, for instance, vulval swelling and vaginal discharge, may help increasing the oestrus detection rate [28].

A poor BCS may have a negative impact on ovarian activity in buffaloes as anoestral buffaloes have lower BCS than buffaloes having oestral cycles [4]. Our study shows that the BCS is not only associated with the interval from calving to first oestrus but also with milk production, as shown previously [29,30]. In the study area, feeding opportunities for grazing buffaloes are limited during December through March. Most of the buffaloes calved during October through December. It is likely that unavailability of forage during the post partum period might have caused reduced BSC and milk production. In fact, good nutritional management in Zebu cows is important to handle the negative energy balance due to milk production [31]. Hayashi *et al.*[32] reported that a decline of nutrient supply as a result of fodder shortage leads to catabolism of body tissue and decreased BCS. Poor BCS of cows not only increases calving to first services interval but also reduces conception rate [9,33].

Anoestral buffaloes had a milk progesterone concentration consistently lower than cyclic buffaloes (<1 ng/ml *vs.* >1 ng/ml). Similarly, in a study of 17 complete post partum periods in Murrah buffaloes in Sri Lanka, plasma progesterone concentrations remained low (<0.25 ng/ml) for a period ranging from 92 to 210 days [34]. McCool *et al.*[35] reported <1 ng/ml plasma progesterone in anoestrus Swamp buffaloes.

Milk progesterone data based on three samples (Day 0, Days 10–12, and Days 22–24) helped us making a clear distinction between pregnant and non-pregnant buffaloes and identification of ovarian cyclicity. One non-pregnant buffalo had a progesterone profile of low, high and low on Day 0, Day 12 and Day 22, respectively. Uçar *et al.*[36] reported progesterone concentrations of 0.97 ± 0.42, 7.99 ± 2.95 and 8.04 ± 2.94 ng/ml on Day 0, Day 11 and Day 21, respectively, of pregnant Anatolian buffaloes. Our results are in agreement with Uçar *et al.*[37] showing progesterone profile of low (<1 ng/ml), high (≥ 1 ng/ml) and high (≥ 1 ng/ml) on Day 0, Days 10–12 and Days 22–24, respectively in pregnant water buffaloes. We showed progesterone profile of low (<1 ng/ml), high (≥ 1 ng/ml) and low (< 1 ng/ml) at Day 0, Days 10–12 and Days 22–24, respectively in cyclic water buffaloes which is in agreement with Qureshi *et al.*[18] who determined progesterone concentrations in defatted milk of dairy buffaloes of 0.30 ± 0.98, 1.43 ± 0.85, 3.29 ± 0.84 and 0.88 ± 0.15 ng/ml on the stage of oestrus, developing corpus luteum, developed corpus luteum and regressing corpus luteum, respectively. Milk progesterone measurement by ELISA was 82-88 % accurate for pregnancy diagnosis in cows [37]. In fact, milk progesterone level on Days 22–24 was used to interpret pregnancy results with 100 % accuracy of non-pregnancy diagnosis in cattle and buffaloes [38]. In our laboratory, 94 % of pregnant cows were accurately detected by using milk progesterone ELISA and 100 % non pregnant cows were detected both by milk progesterone ELISA and radioimmunoassay [16,17]. These findings suggest that a quantitative milk progesterone assay could be used in early pregnancy diagnosis in buffaloes as reported earlier [5,39].

## Conclusions

ELISA was a reliable tool to determine progesterone concentration in milk when investigating post partum ovarian cyclicity in water buffaloes. Oestrus detection needs significant improvement by training farmers to recognise secondary oestus signs and by measuring the progesterone concentration in two samples at 10 days interval if the buffalo cow is not in oestrus at 60 days post partum. This might help the farmers to bred buffaloes in the early post partum period. The nutritional condition throughout the post partum period was important for earlier onset of post partum oestrus in buffaloes.

## Abbreviations

BCS, Body condition score; EDR, Oestrus detection rate; ELISA, Enzyme linked immunosorbent assay; HRP, Horseradish peroxidase; OD, Optical density; SEM, Standard error mean.

## Competing interests

We have no competing interests to declare in relation to this manuscript.

## Authors’ contributions

TAB, MS and JB participated in the study design and coordination. TAB, MS, JB and MFI assisted with sample collection and analysis, completed the statistical analysis and drafted the manuscript. SIK participated in ELISA design and analysis. JUA participated in manuscript preparation and revision. All authors read and approved the final manuscript

## References

[B1] Shah SNH Willemse AH Van de Wiel DFM Descriptive epidemiology and treatment of postpartum anestrus in dairy buffalo under small farm conditions Theriogenol 1990 33 1333 1345 10.1016/0093-691X(90)90051-T

[B2] Uçar M Küçükkebapçi M Gündoğan M Saban E Using milk progesterone assay at the time of oestrus and post-mating for diagnosing early pregnancy in Anatolian water buffaloes Turk J Vet Anim 2004 28 513 518

[B3] Presicce GA Bella A Terzano GM De Santis G Senatore EM Postpartim ovarian follicular dynamics in primiparous and pluriparous Mediterranean Italian Buffaloes (Bubalus bubalis) Theriogenol 2005 63 1430 1439 10.1016/j.theriogenology.2004.07.003 15725449

[B4] Honparkhe M Singh J Dadarwal D Dhaliwal GS Kumar A Estrus induction and fertility rates in response to exogenous hormonal administration in postpartum anestrous and subestrous bovines and buffaloes J Vet Med Sci 2008 70 1327 1331 10.1292/jvms.70.1327 19122399

[B5] Gupta M Prakash BS Milk progesterone determination in buffaloes post insemination Br Vet J 1990 146 563 570 10.1016/0007-1935(90)90061-7 2271913

[B6] Uçar M Küçükkebapçi M Çelebi M Akalin HN Use of milk progesterone assay and rectal palpation to monitor postpartum anoestrus and effect of PRID-PMSG treatment in Murrah buffaloes Turk J Vet Anim 2002 26 1389 1393

[B7] Usmani RH Ahmad M Inskeep EK Dailey RA Lewis PE Lewis GS Uterine involution and postpartum ovarian activity in Nili-Ravi buffaloes Theriogenol 1985 24 435 448 10.1016/0093-691X(85)90050-0 16726098

[B8] Younis M Abass HI Essawy GS Otteifa AM Essawy SA Fadaly M Diagnostic laboratory tests to verify ovulation occurrence with evaluation of accuracy of rectal palpation in buffaloes Egypt J Anim Prod 1994 31 443 451

[B9] Shamsuddin M Bhuiyan MMU Sikder TK Sugulle AH Chanda PK Alam MGS Galloway D Constraints limiting the efficiency of artificial insemination of cattle in Bangladesh. In: Proceedings of a final Research Co-ordination Meeting. Organized by the Joint FAO/IAEA Division of Nuclear Techniques in Food and Agriculture, 10–14 May Uppsala Sweden; 1999 1999 9 28

[B10] Bhalaru SS Tiwana MS Dhillon JS Note on the effect of loss/gain in body weight at insemination on fertility in lactating buffaloes Indian J Anim Sci 1982 52 1232 1234

[B11] Bolaños JM Molina JR Forsberg M Effect of blood sampling and administration of ACTH on cortisol and progesterone levels in ovariectomized zebu cows (Bos indicus) Acta Vet Scand 1997 38 1 7 9129341 10.1186/BF03548502 PMC8057030

[B12] Bhalaru SS Tiwana MS Singh N Effect of body condition at calving on subsequent reproductive performance in buffaloes Indian J Anim Sci 1987 57 33 36

[B13] Ribeiro HFL Andrade VJ Marques AP Vale WG Effect of body condition at parturition on the interval to first postpartum estrus in buffaloes Rev Bras Med Vet 1997 19 213 218

[B14] Rasmussen FE Wiltbank WC Christensen JO Grummer RR Effects of fenprostalene and estradiol-17 beta benzoate on parturition and retained placenta in dairy cows and heifers J Dairy Sci 1996 79 227 234 10.3168/jds.S0022-0302(96)76355-5 8708084

[B15] Wiltbank MC Gümen A Sartori R Physiological classification of anovulatory conditions in cattle Theriogenol 2002 57 21 52 10.1016/S0093-691X(01)00656-2 11775971

[B16] Shamsuddin M Bhuiyan MMU Chanda PK Alam MGS Galloway D Radioimmunoassay of milk progesterone as a tool for fertility control in smallholder dairy farms Trop Anim Health Prod 2006 38 85 92 10.1007/s11250-006-4249-z 17405632

[B17] Khan AHMSI, Department of Surgery and Obstetrics, Bangladesh Agricultural University, Mymensingh, Bangladesh; . Development of milk progesterone ELISA and its application at AI field services in cattle PhD Thesis 2008

[B18] Qureshi MS Habib G Nawab G Siddiwqui MM Ahmad N Samad HA Milk progesterone profiles in various reproductive states in dairy buffaloes under field conditions Proc Natl Sci Counc Repub China B 2000 24 70 75 10809083

[B19] Ali A Fahmy S Ovarian dynamics and milk progesterone concentrations in cycling and non-cycling buffalo-cows (Bubalus bubalis) during ovsynch program Theriogenol 2007 68 23 28 10.1016/j.theriogenology.2007.03.011 17482252

[B20] Murray RD Prokash BS Jailkhani S Madan ML The determination of progesterone in whole and skim milk during oestrous cycle of Murrah buffalo using an enzyme-linked assay and portable plate reader Indian Vet J 1990 67 509 516

[B21] Farin PW Slenning BD Managing reproductive efficiency in dairy herds 2001 WB Saunders, Philadelphia 255 277

[B22] Anon Minitab Statistical Software 2000 Minitab Inc. State College, PA, USA

[B23] Barkawi AH Shafie MM Mekawy Y Aboul-Ela MB The use of serum and milk progesterone concentration to monitor post-partum ovarian activity in Egyptian buffaloes Buffalo J 1986 2 125 134

[B24] Jainudeen MR Reproduction in water buffalo In:Current Therapy in Theriogenology Edited by Morrow DA. Philadelphia: WB Saunders;1986:443-449

[B25] Kamboj M Prakash BS Relationship of progesterone in plasma and whole milk of buffaloes during cyclicity and early pregnancy Trop Anim Health Prod 1993 25 185 192 10.1007/BF02236239 8236496

[B26] Abdalla EB Improving the reproductive performance of Egyptian buffalo cows by changing the management system Anim Reprod Sci 2003 75 1 8 10.1016/S0378-4320(02)00225-7 12535580

[B27] Usmani RH Dailey RA Inskeep EK Effects of limited suckling and varying prepartum nutrition on postpartum reproductive traits of milked buffaloes J Dairy Sci 1990 73 1564 1570 10.3168/jds.S0022-0302(90)78826-1 2384619

[B28] Danell B Clinical and endocrine investigations of the sexual cycle. In: Oestrous Behaviour, Ovarian Morphology and Cyclical Variation in Follicular System and Endocrine Pattern in Water Buffalo Heifers PhD Thesis 1987 Swedish University of Agricultural Sciences, Uppsala 19 53

[B29] Gulati SK Garg MR Serashia PL Scott TW Enhancing milk quality and yield in the dairy cow and buffalo by feeding protected nutrient supplements Asia Pac J Clin Nutr 2003 12(Suppl) S61

[B30] Perera BM Reproductive cycles of buffalo Anim Reprod Sci 2011 124 194 199 10.1016/j.anireprosci.2010.08.022 20869822

[B31] Bolaños JM Forsberg M Kindahl H Rodriguez-Martinez H Biostimulatory effects of oestrus cows and bulls on resumption of ovarian activity in postpartum anestrous Zebu (Bos Indicus) cows in the humid tropics Theriogenol 1998 49 629 636 10.1016/S0093-691X(98)00013-2 10732041

[B32] Hayashi Y Shah S Shah SK Kumagai H Dairy production and nutritional status of lactating buffalo and cattle in small-scale farms in Terai, Nepal LRRD 2005: 17 http://www.lrrd.org/lrrd17/6/haya17064.htm

[B33] Ferguson JD Body Condition Scoring 2002 http://www.txanc.org/ proceedings/2002/Body Condition Scoring.pdf

[B34] Perera BMAO de Silva LNA Kuruwita VY Karunaratne AM Postpartum ovarian activity, uterine involution and fertility in indigenous buffaloes at a selected village location in Sri Lanka Anim Reprod Sci 1987 14 115 127 10.1016/0378-4320(87)90091-1

[B35] McCool CJ Townsend MP Wolfe SG Entwistle KW Endrocrinological studies on pregnancy, postpartum anoestrus and seasonal variation of ovarian activity in the Australian swamp buffalo cow Buffalo J 1987 1 67 72

[B36] Uçar M Küçükkebapçi M Gündoğan M Saban E Using milk progesterone assay at the time of oestrus and post-mating for diagnosing early pregnancy in Anatolian water buffaloes Turk J Vet Anim Sci 2004 28 513 518

[B37] Noakes DE Aurther GH, Noakes DE, Pearson H, Parkinson TJ, Saunders WB Pregnancy and its diagnosis Veterinary Reproduction and Obstetrics 1999 WB Saunders, London 63 109

[B38] Kaul V Prakash BS Accuracy of pregnancy / non pregnancy diagnosis in zebu and crossbred cattle and Murrah buffaloes by milk progesterone determination post insemination Trop Anim Health Prod 1994 26 187 192 10.1007/BF02241083 7809994

[B39] Capparelli R Iannelli D Bordi A Use of monoclonal antibodies for radioimmunoassay of water buffalo milk progesterone J Dairy Res 1987 54 471 477 10.1017/S002202990002567X 3693628

